# Supplementary material for: Modeling driver cells in developing neuronal networks
Source: PLoS Comput Biol. 2018 Nov 2;14(11):e1006551. doi: 10.1371/journal.pcbi.1006551 (PMC6235603; doi:10.1371/journal.pcbi.1006551)
Supplement: S1 Text — (PDF) [file pcbi.1006551.s001.pdf]

Text S1: Experiment - Effect of cell stimulation on the GDP width

In this paper cell-network interaction has been usually measured in terms of the change in the GDP frequency induced by single neuron stimulation. However, we have also verified in the experimental set-up, if single-handedly stimulated drivers could produce a change in the width of the GDP, i.e. in its duration in time. In order to define this width we firstly proceeded to identify GDP events: i) we calculated the average calcium trace (filtered with a Gaussian filter of 0.7 s of width to get rise of the noisy component) as a proxy of the population activity; ii) we identified the local maxima of the negative value of the FURA intensity trace, subject to the conditions that (a) only peaks separated by at least 1 s are considered; (b) the prominence of each peak must be larger than 0.1. The prominence is usually defined in signal processing as the height of the signal above a reference level defined as the highest minimum of the two intervals identified by the imaginary line which starts at the peak and extends towards left and right until it touches the signal again. Fig. S2 (a) displays on top of a time averaged negative FURA intensity the GDPs denoted as triangles. Finally, the width of each GDP is defined as the width of the corresponding peak at half of its prominence, these are also reported in Fig. S2 (a) as black horizontal segments. We considered the distribution of the GDP widths in the three phases of each experiment: namely, pre-stimulation (or control), stimulation and post-stimulation periods. A boxplot reporting the characteristics of these distributions is shown in Fig. S2 (b) for the case illustrated in Fig. S2 (a). From this specific example is clear that there are no significant differences among the three epochs. Furthermore, we performed a Kolmogorov-Smirnov (KS) test among the distributions of the GDP widths in the three phases for each considered experiment, the KS test (with a threshold at 5 % significance level) showed that these distributions never reveal significant modifications from one period to the other in any of the experiments. Finally, with the aim of identifying possible effects on GDP width that cannot be identified at single experiment level, we also calculated the pooled distribution of GDP widths in all experiments during the three epochs (see Fig. S2 (c)). These distributions also show no differences (KS-test) This analysis clearly indicates that cell stimulation mainly affects the frequency of occurrence of GDPs, but not their width.
