# Supplementary material for: Modeling driver cells in developing neuronal networks
Source: PLoS Comput Biol. 2018 Nov 2;14(11):e1006551. doi: 10.1371/journal.pcbi.1006551 (PMC6235603; doi:10.1371/journal.pcbi.1006551)
Supplement: S2 Text — (PDF) [file pcbi.1006551.s002.pdf]

## Text S2: Experiment - Dependence of the results on the stimulation frequency

In order to demonstrate that the absence of any straightforward relationship between the stimulation frequency $\nu_S = \{1/IGI, 0.5Hz, 1Hz\}$ of the driver cells and the induced responses at the network level in the experiments, we performed the following tests. Firstly, we considered all the 30 successful cases in which driver cells were identified in our data set, and we divided these cases into 3 groups corresponding to the three values of the employed stimulation frequency $\nu_S$. These 3 groups are equally populated (33 % of cases for each $\nu_S$), therefore the identification of a driver cell was not related to the specific employed $\nu_S$.

Furthermore, we tested the possibility that there is a correlation between the value of $\nu_S$ and the observed modification of the GDP frequency. Therefore, we performed a paired t-test of the median IGI before and during stimulation protocol in each of the 3 $\nu_S$ to see whether there is a clear trend in the change of the median value for some of the studied frequencies. However, as shown in Fig. S3, none of the studied frequencies is associated to a systematic slow down or acceleration of the GDPs (paired t-test, p-value $< 0.05$).
