# Supplementary material for: Modeling driver cells in developing neuronal networks
Source: PLoS Comput Biol. 2018 Nov 2;14(11):e1006551. doi: 10.1371/journal.pcbi.1006551 (PMC6235603; doi:10.1371/journal.pcbi.1006551)
Supplement: S3 Text — (PDF) [file pcbi.1006551.s003.pdf]

Text S3: Model - Population Burst Variability

We would like to understand if in the model there is a noticeable difference in the synchronized events occurring during stimulation periods with respect to non-stimulation epochs. It is evident from Fig. S9 (a) and (d) that the stimulation of a driver influences not only the frequency of population bursts, but also their variability. In order to measure the PB variability we measured the number of neurons recruited in each burst. In particular, by applying an approach similar to the one considered in [1], we defined a N dimensional vector for each one of the M population bursts (shown in Fig. S9 (a) and (d)) whose i-th entry is 1 if the $i-th$ neuron participated in that population peak and 0 otherwise. For each possible couple of these M vectors we estimated the Jaccard similarity index thus constructing a $M \times M$ matrix, which quantifies the similarity between the population burst events. At first glance it is clear that the PBs can be distinguished in at least in two groups: events with high and low neuronal participation. Similarly to what reported in [1], the large events see the participation of almost all the active neurons, that in control conditions are 78 as reported in Fig. 3 (c) in the main text. In order to discriminate among the two kind of events and to identify the involved neurons we applied a classical clustering algorithm to the similarity matrix, namely a k-means algorithm with $k = 2$.

We observed that the ratio between the number of events in each group indeed changed during the stimulation. In particular the stimulation of the LC driver el_2 (shown in Fig. S9 (a)) induces a decrease in the number of PBs associated with a drastic increase of the number N_H of PBs with a high number of participating neurons. While the stimulation of the LC driver el_1 reported in Fig. S9 (c) induces an increase of the PB frequency together with the number N_L of low populated bursts. Furthermore, the group of neurons involved in the large synchronization events does not vary substantially from one event to the other, while the neurons active during the smaller events are much more heterogeneous (as shown in Fig. S9 (b) and (e)). In order to better understand the origin of this behaviour we have considered the availability of synaptic resources of the stimulated neuron, measured in terms of the average fraction X^{OUT} of synaptic transmitters in the recovery state associated to its efferent synapses, for a definition see Eq. (8) in *Methods*. From Fig. S9 (c) and (f) it is evident that longer IGI allow the neuron to recover large part of its synaptic resources and to X^{OUT} to saturate to some maximal value. In this case the PBs were maximally populated. On the other hand, when the synaptic resources had not time to completely recover the corresponding PBs were poorly populated. It is interesting to note that in the experiments performed in the EC (see Fig .1 (b) in [1]) a large synchronization event is usually followed by smaller ones, quite similarly to what reported in Fig. S9 (a) and (d) for the model. This could represent an indirect indication that the synaptic resources could be also at the origin also to the population burst dichotomy reported in [1]. To render these observations more quantitative we considered the number of events N_H (N_L) with a high (low) number of participating neurons for each stimulation period. Then we plotted the ratio N_H/N_L as a function of the average PB frequency measured in each considered period. As one can see in Fig. S9 (g), this ratio is negatively correlated with the PB frequency (Spearman $\rho = -0.68$, p-value < 0.001), meaning that for high PB frequency, there is a higher chance to elicit a poorly populated PB. This effect is reminiscent of the mechanism leading to the occurrence of Aborted Burst; i.e. network synchronization events occurring at high frequency do not give time to the synapses to recover their optimal condition and therefore not all the active neurons are able to fire in the following synchronization event. This effect is not exclusive of driver LC cells as we have also verified for hub drivers (see red symbols in Fig S9 (g)).

References

1. Mòdol L, Sousa VH, Malvache A, Tressard T, Baude A, Cossart R. Spatial Embryonic Origin Delineates GABAergic Hub Neurons Driving Network Dynamics in the Developing Entorhinal Cortex. Cerebral Cortex. 2017;27(9):4649–4661.
