# Supplementary material for: Modeling driver cells in developing neuronal networks
Source: PLoS Comput Biol. 2018 Nov 2;14(11):e1006551. doi: 10.1371/journal.pcbi.1006551 (PMC6235603; doi:10.1371/journal.pcbi.1006551)
Supplement: S4 Text — (PDF) [file pcbi.1006551.s004.pdf]

Text S4: Model - Robustness of the results to noise

Channel noise is the dominant intrinsic noise source of neurons causing variability in the timing of action potentials and inter-spike intervals (ISI) [1], we therefore decided to focus on this noise source.

To investigate the robustness of our findings against the effect of noise we considered noise strengths Δ ranging in the interval [0.075 : 0.75] mV corresponding to variation from 5% to 50 % of the threshold reset distance, namely $D_V = V_{\rm th} - V_{\rm r} = 1.5$ mV. For details on the implementation of the noisy dynamics see *Definition of the model* in *Methods.* We found that the population burst dynamics is always present for the whole range of explored noise.

To investigate if the composition of the clique of functional hubs is altered by the presence of noise we followed the same approach described in the sub-section *LC drivers impact hub neurons* to verify the impact of the stimulation of a LC driver on the clique (see also Fig. 6 (b-c)). As shown in Fig. S11 (a) for noise amplitudes up to $\Delta = 0.15$ mV (corresponding to 10% of D_V) the clique composition is not affected at all by the presence of noise. Furthermore, up to $\Delta = 0.6$ mV the only effect of noise is preserved apart for ih_2. However, the removal of this hub from the network had already a limited influence on the PB dynamics even in the deterministic case as shown in Fig. 3 (a) in the main text. Only, for extremely large noise amplitudes (namely, $\Delta \geq 0.75$ mV) a new driver cell can be identified. Moreover, we also considered the effect of the noise on the composition and the number of the group of driver LC cells, which have identified by following the procedure described in Section *Impact of single neuron stimulation and deletion on the network dynamics* and whose results are displayed in Fig. 3 (b) in the main text. As shown in Fig. S11 (b) up to $\Delta = 20\% D_V$ the driver LC cells essentially correspond to the ones identified in the absence of noise. Only for quite strong noise, larger than Δ =0.4 mV, we observed the emergence of new LC drivers, which add to the group of the ones identified at zero noise. This effect is probably due to the fact that for large noise it is easier to strongly perturb the coherent population dynamics.

References

1. Faisal AA, Selen LP, Wolpert DM. Noise in the nervous system. Nature reviews neuroscience. 2008;9(4):292.
