# Supplementary material for: Modeling driver cells in developing neuronal networks
Source: PLoS Comput Biol. 2018 Nov 2;14(11):e1006551. doi: 10.1371/journal.pcbi.1006551 (PMC6235603; doi:10.1371/journal.pcbi.1006551)
Supplement: S5 Text — (PDF) [file pcbi.1006551.s005.pdf]

Text S5: Model - Interplay between depression and facilitation for the control of network synchronization

The crucial relevance of the short term synaptic plasticity for network synchronization has been extensively analyzed in the sub-section *Synaptic resources for population bursts* by mainly focusing on aborted bursts. Here we analyze more in details the role played by depression and facilitation for the emergence of PBs. In particular, as an example we consider the SNS experiment performed on the excitatory LC driver el_1, which is directly connected to the inhibitory driver hub ih_1 (as clarified by the sketch reported in Fig. 3 (g) of the main text).

The results of this SNS experiment are shown in Fig. S12. During current stimulation el_1 fires more and therefore the connection between el_1 and ih_1 is subject on one side to synaptic depression measured by the variable X_{ih_1,el_1} and on the other hand to facilitation controlled by the variable u_{ih_1,el_1}. The time average of these two variables is reported in Fig. S12 (a) as a function of the stimulation current revealing an opposite behaviour of synaptic depression and facilitation. As shown in Fig. S12 (b), the combination of these two competing mechanisms results in a non monotonic behavior of the time average of the synaptic variable Y_{ih_1,el_1}, which is indeed the quantity controlling the synaptic plasticity of the connection. In particular, we observe a maximum at an intermediate value of the stimulation current $I_{el_1}^{stim}$ (Fig. S12 (b)). This effect is due to the fact that the variable Y_{ih_1,el_1} is increased at each spikes delivered by el_1 by the amount $u_{ih_1,el_1}X_{ih_1,el_1}$ (see Eqs. (2),(4),(7) in Section it Methods). The behavior of the synaptic variable Y_{ih_1,el_1} is mirrored by the trend of the average firing frequency of ih_1, which also shows a maximum (see Fig. S12 (c)).

For low stimulation currents ($I_{el_1}^{stim} \leq 15.3$ mV), the SNS of eh_1 has a really limited effect on the activity of ih_1 and consequently on the network activity (see Fig. S12 (c-e)). For larger currents, when the connection between el_1 and ih_1 becomes sufficiently strong, the inhibitory action of ih_1 on the functional hub eh_3 becomes effective and leads to a decreases in the number of PBs and to an arrest of the collective dynamics for $I_{el_1}^{\rm stim} \simeq 15.5$ mV. Quite significantly, the PB activity restarts for $I_{el_1}^{\rm stim} \simeq 15.73$ mV when Y_{ih_1,el_1} decreases due to the prevalence of depression and at the same time the firing frequency of ih_1 exhibits a relative minimum (Fig. S12 (b),(d)).

As it is shown in Fig. S12 (e) the number of PBs emitted by the network during the current stimulation of el_1 essentially follows the behavior of the firing frequency of the functional hub eh_3 whose firing dictates the bursting deliver in the network, as explained in Section *Functional clique of excitatory and inhibitory neurons.*

In conclusion, we have shown that the current stimulation of the driver LC cell el_1 is able to control the network bursting through the tuning of the strength of the plastic connection between driver LC and hub cells.
