# Supplementary material for: Modeling driver cells in developing neuronal networks
Source: PLoS Comput Biol. 2018 Nov 2;14(11):e1006551. doi: 10.1371/journal.pcbi.1006551 (PMC6235603; doi:10.1371/journal.pcbi.1006551)
Supplement: S1 Table — Spike time delays ΔT between two successive firing of the neurons forming the functional clique along the main and secondary route leading to bursting. Neurons eh3 and eh4 are assumed to fire essentially at the same time, since eh4 fires almost immediately after eh3 within 0.03 − 0.04 ms. Notice that eh1 is the only driver hub cell firing twice before a PB: the two routes could be distinguished by the time occurrence of the second spike of eh1 and this event is denoted by an asterisk in the table. The arrows indicate the order of firing in the sequence. (PDF) [file pcbi.1006551.s018.pdf]

Main Route		Secondary Route	
Firing Neurons	ΔT (ms)	Firing Neurons	ΔT (ms)
$eh_1 \to ih_1$	3.4 ± 0.2	$eh_1 \to ih_1$	3.3 ± 0.1
$ih_1 \to ih_2$	7 ± 2	$ih_1 \to ih_2$	9 ± 4
$ih_2 \to eh_2$	37 ± 2	$ih_2 \to eh_2$	33 ± 4
$eh_2 \to eh_1^*$	5 ± 2	$eh_2 \to (eh_3 - eh_4)$	6.6 ± 0.6
$eh_1^* \to (eh_3 - eh_4)$	1.5 ± 1.0	$(eh_3 - eh_4) \to eh_1^*$	0.2 ± 0.2

Table 1. Model - Routes leading to PBs Spike time delays ΔT between two successive firing of the neurons forming the functional clique along the main and secondary route leading to bursting. Neurons eh_3 and eh_4 are assumed to fire essentially at the same time, since eh_4 fires almost immediately after eh_3 within $0.03 - 0.04$ ms. Notice that eh_1 is the only driver hub cell firing twice before a PB: the two routes could be distinguished by the time occurrence of the second spike of eh_1 and this event is denoted by an asterisk in the table. The arrows indicate the order of firing in the sequence.
